# Detection of VIM-1-Producing Enterobacter cloacae and Salmonella enterica Serovars Infantis and Goldcoast at a Breeding Pig Farm in Germany in 2017 and Their Molecular Relationship to Former VIM-1-Producing *S.* Infantis Isolates in German Livestock Production

**DOI:** 10.1128/mSphere.00089-19

**Published:** 2019-06-12

**Authors:** Nicole Roschanski, Sead Hadziabdic, Maria Borowiak, Burkhard Malorny, Bernd-Alois Tenhagen, Michaela Projahn, Annemarie Kaesbohrer, Sebastian Guenther, Istvan Szabo, Uwe Roesler, Jennie Fischer

**Affiliations:** a Institute for Animal Hygiene and Environmental Health, Freie Universitaet Berlin, Berlin, Germany; b Department of Biological Safety, German Federal Institute for Risk Assessment, BfR, Berlin, Germany; c Institute of Veterinary Public Health, University of Veterinary Medicine, Vienna, Austria; Escola Paulista de Medicina/Universidade Federal de São Paulo

**Keywords:** *Salmonella*, antimicrobial resistance, carbapenem resistance, carbapenemases, pigs, transmission

## Abstract

In 2011, VIM-1-producing Salmonella enterica serovar Infantis and Escherichia coli were isolated for the first time in four German livestock farms. In 2015/2016, highly related isolates were identified in German pig production. This raised the issue of potential reservoirs for these isolates, the relation of their mobile genetic elements, and potential links between the different affected farms/facilities. In a piglet-producing farm suspicious for being linked to some *bla*_VIM-1_ findings in Germany, fecal and environmental samples were examined for the presence of carbapenemase-producing Enterobacteriaceae and *Salmonella* spp. Newly discovered isolates were subjected to Illumina whole-genome sequencing (WGS) and S1 pulsed-field gel electrophoresis (PFGE) hybridization experiments. WGS data of these isolates were compared with those for the previously isolated VIM-1-producing *Salmonella* Infantis isolates from pigs and poultry. Among 103 samples, one *Salmonella* Goldcoast isolate, one *Salmonella* Infantis isolate, and one Enterobacter cloacae isolate carrying the *bla*_VIM-1_ gene were detected. Comparative WGS analysis revealed that the *bla*_VIM-1_ gene was part of a particular Tn*21*-like transposable element in all isolates. It was located on IncHI2 (ST1) plasmids of ∼290 to 300 kb with a backbone highly similar (98 to 100%) to that of reference pSE15-SA01028. SNP analysis revealed a close relationship of all VIM-1-positive *S*. Infantis isolates described since 2011. The findings of this study demonstrate that the occurrence of the *bla*_VIM-1_ gene in German livestock is restricted neither to a certain bacterial species nor to a certain *Salmonella* serovar but is linked to a particular Tn*21*-like transposable element located on transferable pSE15-SA01028-like IncHI2 (ST1) plasmids, being present in all of the investigated isolates from 2011 to 2017.

**IMPORTANCE** Carbapenems are considered one of few remaining treatment options against multidrug-resistant Gram-negative pathogens in human clinical settings. The occurrence of carbapenemase-producing Enterobacteriaceae in livestock and food is a major public health concern. Particularly the occurrence of VIM-1-producing *Salmonella* Infantis in livestock farms is worrisome, as this zoonotic pathogen is one of the main causes for human salmonellosis in Europe. Investigations on the epidemiology of those carbapenemase-producing isolates and associated mobile genetic elements through an in-depth molecular characterization are indispensable to understand the transmission of carbapenemase-producing Enterobacteriaceae along the food chain and between different populations to develop strategies to prevent their further spread.

## Abstract (precis)

Carbapenems are considered one of few remaining treatment options against multidrug-resistant Gram-negative pathogens in human clinical settings. The occurrence of carbapenemase-producing Enterobacteriaceae in livestock and food is a major public health concern. Particularly the occurrence of VIM-1-producing *Salmonella* Infantis in livestock farms is worrisome, as this zoonotic pathogen is one of the main causes for human salmonellosis in Europe. Investigations on the epidemiology of those carbapenemase-producing isolates and associated mobile genetic elements through an in-depth molecular characterization are indispensable to understand the transmission of carbapenemase-producing Enterobacteriaceae along the food chain and between different populations to develop strategies to prevent their further spread.

## INTRODUCTION

Carbapenems are among the few remaining treatment options for infections caused by multidrug-resistant Gram-negative bacteria. Emergence of bacteria with acquired resistance against carbapenems in human clinical settings is a major public health issue (1). The discovery of VIM-1 carbapenemase-producing Escherichia coli and Salmonella enterica subsp. *enterica* serovar Infantis (*S.* Infantis) in two German pig (*Salmonella* isolates R25 and R27 and E. coli isolates R29 and R178) and one chicken-fattening farm (*Salmonella* isolate R3) in 2011 (2, 3) raised concerns about the spread of this resistance gene in livestock and the development of a new reservoir for the resistance to these last-line antibiotics.

These first findings triggered active monitoring programs in Germany and the European Union (CID 2013/652/EU) (4, 5). Additionally, samplings as well as focused analyses of isolates submitted to the German National Reference Laboratories for *Salmonella* (NRL-Salmonella) and for Antimicrobial Resistance (NRL-AR) are carried out to detect further carbapenemase-producing Enterobacteriaceae (CPE) in food and livestock. Data related to CPE findings are continuously analyzed to carefully monitor the occurrence of these CPE along the food chain. These efforts lead to the recovery of further VIM-1-producing *Salmonella* and E. coli isolates in German livestock and food. Retrospective investigation of bacterial cultures originating from the studies in 2011 identified one additional VIM-1-producing *S*. Infantis isolate (isolate V363) in a sample from a third pig-fattening farm as well as one serologically rough *S.* Infantis isolate (isolate G-336-1a) from a second chicken fattening farm (6, 7). Analysis of *Salmonella* isolates from routine diagnostics revealed two additional *S*. Infantis isolates from minced pork (15-SA01028) in 2015 and from a sick piglet in 2016 (16-SA00749) (8, 9), respectively. This indicated that VIM-1-producing *Salmonella* isolates were still present in German pig production. Three of the six affected livestock farms were additionally positive for E. coli strains carrying *bla*_VIM-1_. The *bla*_VIM-1_ gene was located on an ∼300-kb IncHI2 plasmid in the *Salmonella* isolates (sequence of plasmid pRH-R27 [GenBank accession number LN555650.1] and plasmid pSE15-SA01028 [GenBank accession number CP026661.1]) (3, 7–10). All but one E. coli isolate of sequence type 131 (ST131) belonged to a certain E. coli ST88 clone. These E. coli isolates harbored the *bla*_VIM-1_ gene on smaller plasmids (180 to 230 kb) (6, 7, 11). One of the pig-fattening farms (S2, with highest prevalence of VIM-1-producing *Salmonella* and E. coli) positive in 2011 was intensively sampled and examined 4 years after the first finding. Despite intensive attempts, no VIM-1-positive isolate could be detected. Evaluation of farm-specific keeping and management parameters revealed that disinfection measures were still at a very high level, but the current pigs originated from a different pig breeding farm (12).

Moreover, in 2016, a VIM-1-producing E. coli ST10 strain was isolated in Germany from retail seafood originating from Italy (13). The characteristics of the *bla*_VIM-1_-associated mobile genetic elements from this isolate shared no similarities with those of the VIM-1-positive isolates from the German livestock sector. In this case, the *bla*_VIM-1_-harboring class 1 integron carried further *aacA4*, *aph(3′)-XV*, *aadA1*, and *catB2* gene cassettes in its variable region and was associated with a Tn*3*-like transposon on an IncY plasmid. In the livestock isolates, the *bla*_VIM-1_-harboring class 1 integron carried further *aacA4*-*aadA1* gene cassettes and was associated with a Tn*21*-like transposon on IncHI2 plasmids.

Carbapenems are not licensed for use in livestock. However, administration of any other beta-lactam antibiotic or other antimicrobial classes with resistance genes genetically linked to carbapenemase-encoding genes might trigger the selection of CPE and the spread of the respective mobile genetic elements. This may have contributed to the recurrent detection of *bla*_VIM-1_-containing E. coli as well as *S.* Infantis over a period of 5 years (2011 to 2016). However, the sporadic occurrence of CPE, their low detection rates accompanied by the complexity of trade routes within the German pig production system, complicates trace-back approaches. Nevertheless, based on analysis of available background information of affected farms we identified a piglet-producing farm (N2) suspicious for being linked to at least two previously affected pig farms (including S2) and collected fecal and environmental samples on this farm. In order to characterize the mobile genetic elements and phylogeny of the isolates involved in this occurrence, we carried out in-depth molecular analysis using whole-genome sequencing (WGS) of isolates recovered from this piglet-producing farm and compared the data with sequence data from all seven VIM-1-positive *S.* Infantis isolates previously described.

## RESULTS

### Phenotypic and genotypic characterization of isolates and *bla*_VIM-1_-associated mobile genetic elements. (i) Isolates from pig breeding farm N2.

Using the modified DIN EN ISO 6579 method, six *Salmonella* isolates were obtained from four samples (Table 1). Isolates were identified by classical serology as either *S*. Infantis (*n* = 1; isolate N2-1) or S. Goldcoast (*n* = 5; isolates N2-2 to N2-6). Results were confirmed using the SISTR Salmonella *in silico* typing tool. The *S.* Infantis isolate N2-1 (collected feces, rearing quarter 1) as well as *S.* Goldcoast isolate N2-2 (boot swab, farrowing barn 4) tested positive for the *bla*_VIM-1_ gene by PCR. Four *S*. Goldcoast isolates, detected in boot swabs (*n* = 2), collected feces (*n* = 1), and manure (*n* = 1), did not carry the *bla*_VIM-1_ gene. All five *S*. Goldcoast isolates belonged to ST358 (Table 1). The *S*. Infantis isolate, like all other previously described VIM-1-positive *S*. Infantis isolates, belonged to ST32. Neither a *bla*_VIM-1_-negative *S*. Infantis isolate nor any other nonmotile *Salmonella* spp. were detected.

**TABLE 1:** Phenotypic and genotypic characteristics of isolates derived from farm N2 and previously described *bla*_VIM-1_-positive *S*. Infantis isolates^*a*^

Isolate	Yr of isolation	Source, farm origin (sample name)	Species or serovar (MLST)	MICs (mg/liter) of ETP, IMP, and MEM (April 2019)	MICs (mg/liter) of FOT and TAZ	Presence of pAmpC- or carbapenemase- encoding genes	Reference
N2-8	2017	Collected feces, swine farm N2 (FD1-SK2)	Enterobacter cloacae	1, 4, 2	>64, >128	*bla*_ACC-1_, *bla*_ACT-7_-like, *bla*_VIM-1_	This study
N2-6	2017	Manure, swine farm N2	*S*. Goldcoast (ST358)	≤0.015, 0.25, 0.006	32, 128	*bla*_ACC-1_	This study
N2-5	2017	Collected feces, swine farm N2 (FD1-SK1)	*S*. Goldcoast (ST358)	≤0.015, 0.25, ≤0.03	≤0.25, 0.5		This study
N2-4	2017	Boot swabs, swine farm N2 (FD1-Sta)	*S*. Goldcoast (ST358)	≤0.015, 0.25, ≤0.03	≤0.25, 0.5		This study
N2-3	2017	Boot swabs, swine farm N2 (A4-Sta)	*S*. Goldcoast (ST358)	≤0.015, 0.25, ≤0.03	≤0.25, 0.5		This study
N2-2	2017	Boot swabs, swine farm N2 (A4-Sta)	*S*. Goldcoast (ST358)	0.5, 4, 2	>64, >128	*bla*_ACC-1_, *bla*_VIM-1_	This study
N2-1	2017	Collected feces, swine farm N2 (FD1-SK2)	*S*. Infantis (ST32)	0.125, 2, 0.5	>64, >128	*bla*_ACC-1_, *bla*_VIM-1_	This study
16-SA00749	2016	Sick piglet, swine farm N1	*S*. Infantis (ST32)	0.25, 4, 2	>64, >128	*bla*_ACC-1_, *bla*_VIM-1_	9
15-SA01028	2015	Minced pork meat	*S*. Infantis (ST32)	0.5, 4, 2	>64, >128	*bla*_ACC-1_, *bla*_VIM-1_	9
G-336-1a	2012	Collected dust, poultry farm G78	*S*. Infantis (ST32) serologically rough	>2, 8, 16	>64, >128	*bla*_ACC-1_, *bla*_VIM-1_	6
V363	2012	Single animal feces, swine farm S3	*S*. Infantis (ST32) nonmotile	2, 8, 4	>64, >128	*bla*_ACC-1_, *bla*_VIM-1_	7
R27	2011	Pooled faces, swine farm S2	*S*. Infantis (ST32) nonmotile	0.25, 4, 0.5	>64, >128	*bla*_ACC-1_, *bla*_VIM-1_	3
R25	2011	Boot swabs, swine farm S1 environment	*S*. Infantis (ST32) nonmotile	0.25, 4, 0.5	>64, >128	*bla*_ACC-1_, *bla*_VIM-1_	3
R3	2011	Collected dust, poultry farm G1	*S*. Infantis (ST32) nonmotile	0.25, 4, 0.5	>64, >128	*bla*_ACC-1_, *bla*_VIM-1_	3

a ETP, ertapenem; IMP, imipenem; MEM, meropenem; FOT, cefotaxime; TAZ, ceftazidime.

One *bla*_VIM-1_-positive Enterobacter cloacae isolate (N2-8) was found through reanalysis of the collected fecal sample harboring the *bla*_VIM-1_-positive *S*. Infantis using nonselective preenrichment followed by a selective cultivation in LB medium supplemented with 1 mg/liter of cefotaxime sodium salt (CTX).

An overview of isolate characteristics is depicted in Table 1. Pulsed-field gel electrophoresis (PFGE) restriction patterns of all previously isolated *S*. Infantis isolates and the recently discovered isolate N2-1 were highly similar (see Fig. S1 in the supplemental material). Likewise, the PFGE restriction patterns of all five *S*. Goldcoast isolates were alike (Fig. S2). MIC testing of isolates harboring the *bla*_VIM-1_ gene revealed a low-level resistance against the tested carbapenems but a high-level resistance against the third-generation cephalosporins tested (Table 1) in contrast to isolates lacking the *bla*_VIM-1_ gene.

10.1128/mSphere.00089-19.1 FIG S1 XbaI-PFGE analysis of all six *bla*_VIM-1_-positive *S*. Infantis described so far and within the scope of this study recovered *S*. Infantis isolate N2-1. Br, molecular size standard *Salmonella* Braenderup strain H9812 (restricted with XbaI). Download FIG S1, PDF file, 0.1 MB. Copyright © 2019 Roschanski et al. 2019 Roschanski et al. This content is distributed under the terms of the Creative Commons Attribution 4.0 International license.

10.1128/mSphere.00089-19.2 FIG S2 XbaI-PFGE analysis of all five *S*. Goldcoast isolates (N2-2 to N2-6) selected in this study and the *bla*_VIM-1_-positive Entereobacter cloacae isolate (N2-8). Br, molecular size standard *Salmonella* Braenderup strain H9812. Download FIG S2, PDF file, 0.1 MB. Copyright © 2019 Roschanski et al. 2019 Roschanski et al. This content is distributed under the terms of the Creative Commons Attribution 4.0 International license.

**FIG 1:**
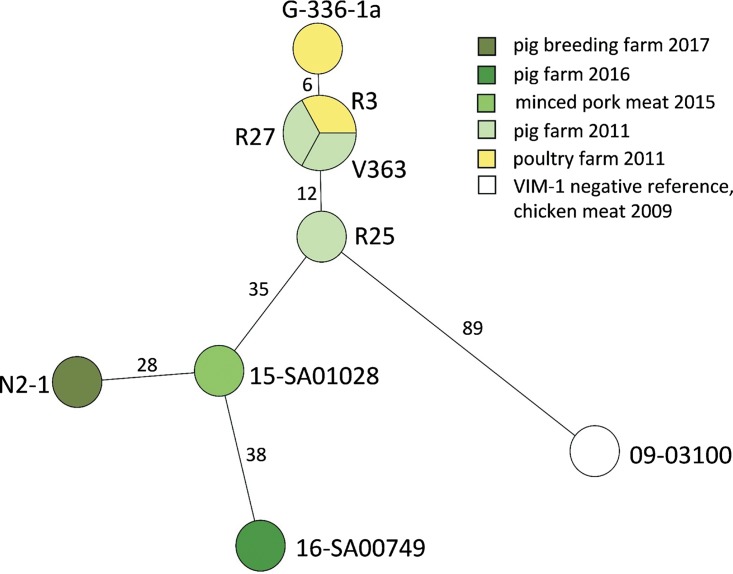
Minimum spanning tree of *bla*_VIM-1_ harboring *S*. Infantis isolates based on genome SNP analysis with the complete genome of *S*. Infantis 15-SA01028. Branches are labeled with the number of SNP differences. Darker greenish colors reflect most recent isolates obtained. Isolate 09-03100 served as the nearest VIM-1-negative *S*. Infantis neighbor for comparison purposes.

**(ii) Phylogeny and SNP analysis of *S*. Infantis isolates.** Mapping of raw reads against the PacBio chromosome sequence of isolate 15-SA01028 and subsequent single nucleotide polymorphism (SNP) analysis revealed 0 to 51 SNPs per chromosome. Isolates from 2011 and 2012 showed fewer SNPs (0 to 18), while their distance to isolates obtained in 2015 to 2017 was 28 to 51 SNPs. All *bla*_VIM-1_-harboring isolates clustered separately with a distance of >89 SNPs to the closest *bla*_VIM-1_-negative *S*. Infantis relative, isolate 09-03100 (14) (Fig. 1 and Table S2).

**FIG 2:**
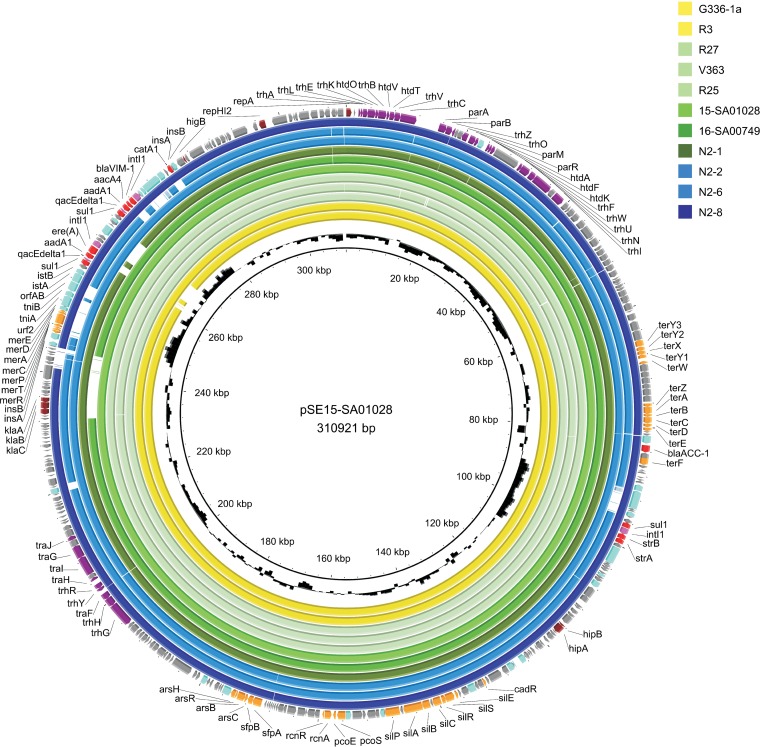
Comparative plasmid maps (BRIG) of isolates from farm N2 and *bla*_VIM-1_-harboring *Salmonella* isolates from previous studies, showing sequence identities of >90% to reference pSE15-SA01028 (GenBank accession number CP026661). Isolates G336-1a and N2-1 lack the In*1516* class 1 integron harboring *ereA* and *aadA1* gene cassettes downstream of the *bla*_VIM-1_-containing class 1 integron. This integron was previously not described on pRH-R27 by Falgenhauer et al. (10). In isolate N2-6, PCR-based sequence gap closure revealed that the whole *bla*_VIM-1_-harboring Tn*21*-like transposon is absent in this plasmid region. Blue circles depict plasmids of *S*. Infantis isolates from pigs, yellow circles of *S*. Infantis from poultry farms, and green circles of *S*. Goldcoast isolates, and the red circle depicts the plasmid of the E. cloacae isolate.

**FIG 3:**
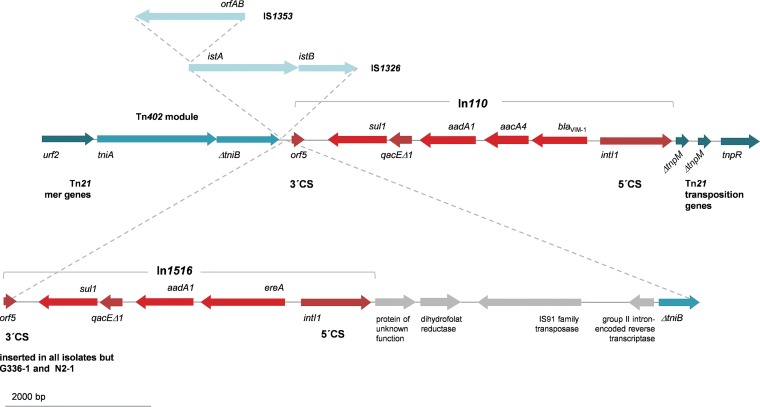
Observed variants of the Tn*21*-like transposon already described by Falgenhauer et al. (10), bearing the *bla*_VIM-1_ gene on a class 1 integron with additional resistance genes *aacA4* and *aadA1* in its variable region. Cyan arrows represent transposon-associated genes, red ones antimicrobial resistance and class 1 integron-associated genes, and gray arrows cryptic DNA sequences. The class 1 integron harboring *ereA* and *aadA1* gene cassettes, downstream of the *bla*_VIM-1_-containing class 1 integron, was previously not described on pRH-R27 by Falgenhauer et al. (10) and was only present in isolates N2-2, N2-8, 16-SA00749, 15-SA01028, V363, R27, R25, and R3.

**(iii) Mobile genetic elements.** S1 PFGE hybridization experiments revealed the presence of *bla*_VIM-1_-harboring 290- to 300-kb plasmids for isolates N2-1 (*S*. Infantis), N2-2 (*S*. Goldcoast), and N2-8 (E. cloacae) (Fig. S2). WGS results confirmed presence of IncHI2 (ST1) plasmids with consensus sequence lengths between 304 and 311 kb and sequence similarities of 98 to 100% to reference plasmid pSE15-SA01028 (Table 2). Detailed comparison of the plasmid sequences, visualized using the BLAST Ring Image Generator (BRIG) (Fig. 2), revealed that the main differences between plasmids are based on structural changes in the *bla*_VIM-1_-harboring Tn*21*-like transposon. In all plasmids the *bla*_VIM-1_ gene was part of an In*110* class 1 integron accompanied by the genes *aacA4* and *aadA1* in its variable region, as previously described for pRH-R27 (10). In p15SA-01028 and all isolates but G336-1 and N2-1, however, an insertion of a further In*1516* class 1 integron harboring an *ereA* and an *aadA1* gene cassette was observed. This additional integron was located upstream of the IS*1326* module and was itself flanked upstream by cryptic coding DNA sequences, comprising several genes commonly found associated with other mobile genetic elements (Fig. 3). Although the *bla*_VIM-1_ gene was not present in the *S*. Goldcoast isolate N2-6 from manure, a 93% identity to pSE15-SA01028 could be observed for the plasmid harbored by this isolate (286.971-bp consensus sequence length, confirmed by an ∼290-kb band in the S1 PFGE [Table 2 and Fig. S2]). In this isolate, absence of the *bla*_VIM-1_ gene is due to the lack of the complete Tn*21*-like transposon in this plasmid region that could be confirmed through closing the plasmid sequence via PCR sequencing at this position.

**TABLE 2:** Occurrence and molecular characteristics of pSE15-SA01028-like plasmids in isolates derived from farm N2 and previously *bla*_VIM-1_-positive isolates from food, pig, and poultry farms in Germany

Isolate	Presence of pAmpC- or carbapenemase- encoding gene(s)	Size (kb) of *bla*_VIM-1_-harboring plasmids^*a*^	Presence of IncHI2 plasmid^*b*^	Total consensus sequence length (bp)	% sequence identity to p15-SA01028	Tn*21-*like variant
N2-8	*bla*_ACC-1_, *bla*_ACT-7_-like, *bla*_VIM-1_	300	IncHI2 (ST1)	307.594	99	As pSE15-SA01028
N2-6	*bla*_ACC-1_		IncHI2 (ST1)	286.971	92	
N2-5				<30	<10	
N2-4				<30	<10	
N2-3				<30	<10	
N2-2	*bla*_ACC-1_, *bla*_VIM-1_	300	IncHI2 (ST1)	310.794	100	As pSE15-SA01028
N2-1	*bla*_ACC-1_, *bla*_VIM-1_	290	IncHI2 (ST1)	304.382	98	Absence of In*1516*^*c*^
16-SA00749	*bla*_ACC-1_, *bla*_VIM-1_	290	IncHI2 (ST1)	298.680	96	As pSE15-SA01028
15-SA01028	*bla*_ACC-1_, *bla*_VIM-1_	300	IncHI2 (ST1)	310.921	100	Reference (CP026661)
G-336-1a	*bla*_ACC-1_, *bla*_VIM-1_	300	IncHI2 (ST1)	305.248	98	Absence of In1516^*c*^
V363	*bla*_ACC-1_, *bla*_VIM-1_	300	IncHI2 (ST1)	310.162	100	As pSE15-SA01028
R27	*bla*_ACC-1_, *bla*_VIM-1_	300	IncHI2 (ST1)	310.871	100	As pSE15-SA01028
R25	*bla*_ACC-1_, *bla*_VIM-1_	300	IncHI2 (ST1)	310.921	100	As pSE15-SA01028
R3	*bla*_ACC-1_, *bla*_VIM-1_	300	IncHI2 (ST1)	310.921	100	As pSE15-SA01028

a Based on S1 restriction and subsequent hybridization.

b Based on CGE batch upload analysis.

c As described by Falgenhauer et al. (10).

## DISCUSSION

In this study, two *bla*_VIM-1_-producing *Salmonella* isolates (one *S*. Infantis and one *S*. Goldcoast) and one *bla*_VIM-1_-producing Enterobacter cloacae isolate were isolated within an investigation of a farrow-to-wean farm suspicious for having connections to two previously pig-associated *bla*_VIM-1_ findings in Germany.

These findings indicate that in 2017 still a source, bearing potential to transfer VIM-1-producing isolates to German pig producers, was present. However, the low number of obtained VIM-1-producing isolates, despite an extensive sampling effort, suggests a very low prevalence of VIM-1-producing isolates in this farm. In-depth molecular analysis using WGS techniques revealed a close relationship of all *bla*_VIM-1_-harboring *S*. Infantis isolates from German livestock recovered until now. The high degree of molecular similarity of isolates and associated mobile genetic elements hints at a connection of affected farms that is not obvious through analysis of available background information, especially for the poultry-related findings. Since *S.* Infantis was detected in five pig-associated cases and two positive chicken-fattening farms, this serovar might play a particular role in the occurrence of *bla*_VIM-1_. However, comparative analysis in this study revealed that *bla*_VIM-1_ in Germany is not restricted to a certain serovar but might be regarded as a long-term multispecies-related occurrence, linked to a certain pSE15-SA01028-like IncHI2 plasmid. All nine *bla*_VIM-1_-positive *Salmonella* isolates as well as the *bla*_VIM-1_-positive E. cloacae isolate harbored this gene on ∼290- to 300-kb IncH12 (ST1) plasmids with a backbone of 96 to 100% similarity to pSE15-SA01028 and linked to a certain Tn*21-*like transposable element. Interestingly, mobility of the Tn*21-*like transposon carrying the *bla*_VIM-1_ gene could be verified through the detection of *S*. Goldcoast N2-6 (isolated from manure) with a plasmid showing 92% identity to pSE15-SA01028 but lacking the complete *bla*_VIM-1_-containing Tn*21*-like transposon. However, since active transposition of Tn*21*-like transposable elements is a replicative mechanism, the loss of the whole transposon has possibly been mediated by homologous recombination involving two flanking IS*1* elements (*insA/insB* [Fig. 2]), as in isolate N2-6, only one *insA/insB* copy is left. Mobility of this transposon was previously also proven through *in vitro* experiments, revealing acquisition of the *bla*_VIM-1_-carrying Tn*21* on an IncI1 plasmid that was colocated in a VIM-1-producing E. coli isolate (6). The sole presence of this certain *bla*_VIM-1_-carrying Tn*21* in any bacterial species or plasmid could be a hint of a potential relation to the *bla*_VIM-1_ findings in German livestock. It is noteworthy that the *ereA-aadA1*-harboring integron downstream of the *bla*_VIM-1_-*aacA4-aadA1* integron was previously not described on pRH-R27 by Falgenhauer et al. (10). This discrepancy might be an artifact from Roche 454 short-read assembly or could be based on loss of this region during the sample preparation for sequencing by Falgenhauer et al. (10). The absence of this region could also be observed for isolates G336-1a and N2-1.

Transfer of the pSE15-SA01028-like IncHI2 plasmids seems to be less efficient, since *in vitro* transfer of the *bla*_VIM-1_ plasmid at least starting from *S*. Infantis is difficult to prove (3, 10). However, the presence of this plasmid in *S*. Goldcoast, E. cloacae, and several E. coli STs (6, 7, 11) unambiguously confirms its transferability *in vivo*.

In addition, the results of the SNP analysis of the VIM-1-producing *S*. Infantis isolates indicate that transfer *in vivo* might be a rare event and that there might have been just a sole acquisition event in one common *S*. Infantis ancestor cell. All eight *bla*_VIM-1_-positive *S*. Infantis isolates show fewer than 51 SNPs (Table S2 and Fig. 1), confirming their close genetic relationship that had already been assumed on the basis of PFGE analysis (3). Moreover, they build a separate cluster (>89 SNPs to the closest relative, 09-03100) within the *S*. Infantis population circulating in German food and livestock (14).

Among this cluster, isolates from 2011/2012 share the lowest number of SNPs (0 to 18 SNPs [Fig. 1 and Table S2]). Assuming in *S.* Infantis a SNP rate at least as high as in S. Typhimurium with 3 to 5 SNPs per year per chromosome (15), this sole transfer event might have occurred indeed shortly before or within the time frame of the first findings of VIM-1-positive Enterobacteriaceae in 2011/2012. In fact, the number of SNPs between the isolates of our study suggests an even higher SNP rate for S. Infantis. For *Salmonella*, disease outbreaks are usually characterized by SNP variations of 0 to 30 and varying within each serovar and clonal lineage (16). Finally, SNP- and plasmid-based analyses in this study indicate that this occurrence of *bla*_VIM-1_ in Germany might be regarded as a single low-level multispecies event that drags on for obviously more than 6 years.

Following the hypothesis of a single transfer event into *S*. Infantis, the acquisition of this VIM-1 metallo-beta-lactamase-encoding plasmid might have been associated with an alteration of *Salmonella* surface antigens leading to the nonserotypable phenotype using the Kaufmann-White scheme, as this is only observed for the *S.* Infantis isolates from 2011/2012. A similar phenomenon was already described for an *S.* Typhimurium strain acquiring a *bla*_VIM-2_ gene. In this case, acquisition of the *bla*_VIM-2_ gene resulted in alterations in micro- and macroscopic morphology, a reduced growth rate, and decreased motility (17). Since *S.* Infantis isolates from 2015 and later show a classical serological *S*. Infantis phenotype, subsequent genetic adaptations might have allowed conquest of serological constraints.

Although no *bla*_VIM-1_-harboring E. coli was detected in this study, recent reports of E. coli harboring highly similar or identical *bla*_VIM-1_-bearing plasmids (6, 11) suggest the potential existence of a low-abundance E. coli subpopulation in the German livestock sector that might be able to pop up through antibiotic treatment like was assumed for the VIM-1-producing E. coli ST88 contamination in pig farm S2 (7, 12).

The detection of the E. cloacae only through reanalysis of samples positive for VIM-1-producing *Salmonella* underlines that the detection of CPE in livestock samples is challenging due to their low prevalence and the low-level expression of carbapenemase-encoding genes under nonselective pressure (MICs of CPE in this study ranged from 0.12 to 4 mg/liter). Low-level expression of the *bla*_VIM-1_ gene in isolates that had not undergone antibiotic selective pressure was already reported for *bla*_VIM-1_ *Salmonella* and E. coli isolates from German livestock and food (3, 13). However, a higher level of carbapenem resistance has been observed through their cultivation in broth supplemented with a carbapenem (3). Differences in MICs between isolates of this study (although harboring a highly similar or identical plasmid) or after reanalysis of isolates several years after their first MIC testing underline that the level of carbapenem resistance might be influenced by different origins and evolution of isolates (reflected through SNP differences) or even isolate handling conditions (different isolation, cultivation, and storing conditions in the different studies). These conditions might affect the expression of the *bla*_VIM-1_ gene or the copy number of the plasmids (e.g., through antibiotic selective pressure).

In human clinical settings in Germany, *bla*_VIM-1_ was first described in 2007 (18); today, it is the second most frequently detected carbapenemase gene in Enterobacteriaceae, with E. cloacae being the main *bla*_VIM-1_ reservoir (19). In contrast, no VIM-1-producing *Salmonella* isolate from humans in Germany has been described so far (20). Currently there is no literature available on *bla*_VIM-1_-harboring plasmids in human clinical isolates in Germany. This raises questions on the role of the E. cloacae isolate N2-8 serving either as one of the recipients that took up the plasmid from *S*. Infantis or as the initial donor of the *bla*_VIM-1_ plasmid. Further studies have to elucidate whether the *bla*_VIM-1_ gene in human clinical E. cloacae isolates is also associated with pSE15-SA01028-like plasmids. Answers to these questions require a “one health” approach, including expanded in-depth comparative analysis, encompassing VIM-1-harboring plasmids and isolates circulating in the human population and the environment in Germany.

Since the first findings of CPE in livestock, a number of studies have reported carbapenemase-producing bacteria with different carbapenemase-encoding genes in food-producing animals, including pigs, and companion animals worldwide (21–26). Whether the globally increasing reports on CPE in livestock and along the food chain are due to increased awareness of this issue followed by intensified screening activities or if this reflects the current emergence of livestock as a new reservoir for CPE, probably triggered through their increased emergence in the human clinical sector, needs to be further investigated.

In Germany, the persistence of a *bla*_VIM-1_ gene located on a Tn*21*-like transposon on a transferable plasmid was shown in recent years. Evaluation of WGS data just allows assumptions on the source of the *bla*_VIM-1_ gene on the farms; the agent that actually served as the initial bacterial source remains unknown. However, *S*. Infantis, which is frequently found in poultry and pigs in Germany and is among the main causes of human salmonellosis in Europe (27, 28), may play a certain role in this case. The fact that the VIM-1-producing *S*. Infantis was isolated in each of the affected farms but is rarely found through screening or retrospective analysis (6, 7) suggests that this serovar serves at least as an appropriate reservoir, stably hosting this particular *bla*_VIM-1_ pSE15-SA01028-like plasmid. Finally, consideration must be given to how to handle the sources of these VIM-1-producing isolates, such as the breeding farm under investigation in this study. Although persistence of CPE in animal surroundings was shown to not be dependent on selective pressure (29), antibiotic treatments such as those with amoxicillin might have triggered their persistence in this occurrence. Particular attention should be paid to detailed analysis of the epidemiological situation on the farm, i.e., to determine in which groups of sows the isolates circulate. It should also be examined whether a source of entry from the environment via feed or other vectors can be identified. Based on these results, specific action should be taken to stop the introduction and circulation of these bacteria within the farm and to minimize the risk of spreading the bacteria to fattening farms. Groups of sows identified to be infected with VIM-1-producing Enterobacteriaceae should be replaced and affected facilities or stables should undergo strict cleaning, disinfection, and hygiene measures. Since *Salmonella* plays a special role in the current infection, a *Salmonella* vaccination program might also be considered to support the eradication of these carbapenemase-producing *Salmonella* strains from the herd.

## MATERIALS AND METHODS

### Sampling at the pig farm.

Sampling of the breeding farm was performed during on-site visitation by the Institute for Animal Hygiene and Environmental Health of Freie Universitaet Berlin. It covered three breeding centers, four gestation stalls, five farrowing barns (each containing approximately 75 farrowing pens), and five rearing units. During the visit, one breeding center and one farrowing barn were not in use. The type and number of samples varied depending on the number of sows/piglets and subunits in each barn (Table S1). In the gestation stalls and the rearing quarters, mostly 10 to 15 sows or weaners were housed per pen. From each of the units approximately five distinct fecal samples were collected and pooled per pen. Samples taken per pen were additionally pooled as shown in Table S1. In the breeding centers and the farrowing barns, where the sows were housed in individual stands, feces from several animals were collected and pooled (Table S1). Boot swab samples were taken from the central corridor of each housing unit. Liquid manure samples were collected from each of the three manure pits.

10.1128/mSphere.00089-19.4 TABLE S1 Overview of the number of samples and the sampled material per barn. Download Table S1, PDF file, 0.1 MB. Copyright © 2019 Roschanski et al. 2019 Roschanski et al. This content is distributed under the terms of the Creative Commons Attribution 4.0 International license.

10.1128/mSphere.00089-19.5 TABLE S2 SNP analysis of *S.* Infantis isolates recovered in this study, compared with *bla*_VIM-1_-positive *S*. Infantis isolates from previous studies. Download Table S2, PDF file, 0.10 MB. Copyright © 2019 Roschanski et al. 2019 Roschanski et al. This content is distributed under the terms of the Creative Commons Attribution 4.0 International license.

### Laboratory analyses of the samples: screening for CPE and *Salmonella* spp. (i) Preenrichment of samples.

All samples were processed within 24 h after sampling. Of each pool of fecal samples, 20 g was inoculated in 180 ml of peptone water (buffered peptone water; Carl Roth GmbH, Karlsruhe, Germany) without any supplement as recommended by the European Union Reference Laboratory for Antimicrobial Resistance (EURL-AR [https://www.eurl-ar.eu/protocols.aspx]). Boot swabs were incubated in 100 ml of buffered peptone water. From each liquid manure sample, 5 g was inoculated in 45 ml of peptone water. After an overnight incubation at 37°C, aliquots of each culture were stored at −80°C for future investigations. All cultures were subjected to CPE detection procedures as follows.

**(ii) DNA preparation, screening, and identification of carbapenemase-encoding genes using PCR.** Each of the incubated peptone water cultures was tested with direct real-time PCR. The DNA preparation as well as the real-time PCR based assay, detecting the carbapenemase genes *bla*_VIM_, *bla*_KPC_, *bla*_NDM_, *bla*_OXA-48_, and *bla*_GES_, were performed as previously described (30). Subsequent bacterial species identification was performed using matrix-assisted laser desorption ionization–time of flight (MALDI-TOF) mass spectrometry (MS) (MALDI Microflex LT and Biotyper database; Bruker Daltronics, Bremen, Germany).

**(iii) Isolation of CPE via incubation on selective agar plates.** The overnight cultures were streaked on (i) ChromID Carba (bioMérieux, France) as well as (ii) MacConkey agar plates containing 1 mg/liter of cefotaxime sodium salt (CTX; Merck KGaA, Darmstadt, Germany). On the following day, MALDI-TOF MS (MALDI Microflex LT and Biotyper database; Bruker Daltonics, Bremen, Germany) was performed from randomly selected colonies (one colony per morphology was analyzed per plate) to determine bacterial species. Subsequent confirmation of the presence of the carbapenemase-encoding gene was determined by real-time PCR as mentioned above (see “DNA preparation, screening, and identification of carbapenemase-encoding genes using PCR”).

**(iv) Isolation of *Salmonella* following a modified DIN EN ISO 6579 protocol adapted to the detection of nonmotile *Salmonella* variants.** As *S.* Infantis with different serological characteristics (serologically rough, nonmotile S. enterica group C with antigenic formula “6,7:-:-” or classically typed as *S.* Infantis 6,7:r:1,5) were found in previously affected pig and poultry farms, a parallel in-depth screening for *Salmonella* was performed. *Salmonella* species isolates were obtained according to ISO 6579:2002+Amd 1:2007, with subsequent serotyping according to the White-Kauffmann-Le Minor scheme (31).

Since the VIM-1-producing *S*. Infantis isolates from 2011/2012 were nonmotile, the DIN EN ISO 6579 protocol was expanded and included both selective enrichment with Rappaport-Vassiliadis medium with soya (RVS-Bouillon) for nonmotile *Salmonella* variants and modified semisolid Rappaport-Vassiliadis medium (MSRV) for motile variants. Furthermore, cultures were incubated for 48 h on xylose-lysine-deoxycholate agar (XLD agar) with and without 1 mg/liter of CTX.

**(v) Detailed analyses of single cultures.** Samples that tested negative for CPE but positive for VIM-1-producing *Salmonella* by using the modified DIN EN ISO 6579 protocol were analyzed a second time. The stored (−80°C) cultures as well as the stored primary overnight cultures were revitalized (1:100) in 5 ml of LB medium containing 1 mg/liter of CTX. The derived cultures were subsequently spread on ChromID Carba plates (bioMérieux, France) and MacConkey agar plates containing 1 mg/liter of CTX and 0.125 mg/liter of meropenem (MEM). Single colonies were selected for species identification using MALDI-TOF MS followed by a CPE status confirmation using real-time PCR, as described in “DNA preparation, screening, and identification of carbapenemase-encoding genes using PCR” above.

### Phenotypic and genotypic characterization of the carbapenemase-producing isolates. (i) Antimicrobial susceptibility testing.

Determination of MICs of the carbapenems ertapenem, meropenem, and imipenem for presumptive CPE was carried out according to Commission Implementing Decision 2013/652/EU on the monitoring and reporting of antimicrobial resistance in zoonotic and commensal bacteria. MICs were determined/redetermined using the broth microdilution method and following guidelines described by the Clinical and Laboratory Standards Institute (CLSI) (32, 33), including, among other antimicrobial substances, the third-generation cephalosporins cefotaxime and ceftazidime and the carbapenems meropenem, ertapenem, and imipenem.

**(ii) PFGE and Southern blot hybridization.** CPE and *Salmonella* isolates underwent XbaI and S1 nuclease restriction of bacterial DNA and subsequent pulsed-field gel electrophoresis (PFGE) (34) using the CHEF-DR III system (Bio-Rad Laboratories GmbH, Munich, Germany) as previously described (35).

To determine the localization of the *bla*_VIM-1_ gene in these strains, Southern blot and hybridization analyses of S1 PFGE gels with a digoxigenin-labeled *bla*_VIM-1_ probe were conducted (35).

### WGS. (i) Library preparation.

All *Salmonella* isolates and isolates harboring a *bla*_VIM-1_ gene were subjected to whole-genome sequencing (WGS) analysis. Liquid LB medium containing 1 mg/liter of CTX was inoculated with a single colony grown on LB agar. Broths were cultivated under shaking conditions (180 to 220 rpm) at 37°C for 14 to 16 h. DNA was isolated using the PureLink genomic DNA minikit (Invitrogen, Carlsbad, CA) followed by preparation of sequencing libraries using the Nextera XT DNA sample preparation kit (Illumina, San Diego, CA) according to the manufacturer’s protocol. Paired-end sequencing was performed in 2 × 251 cycles on the Illumina MiSeq benchtop using the MiSeq Reagent v3 600-cycle kit (Illumina).

**(ii) Comparative analysis of WGS data of isolates and *bla*_VIM-1_-harboring plasmids.** Raw reads were trimmed using trimmomatic (36), and trimmed sequencing data were *de novo* assembled using SPAdes (https://cge.cbs.dtu.dk/services/SPAdes/). Assemblies led to an average contig number of 169 with an average coverage of 67. Contig sequences allowed determination of multilocus sequence type (MLST), plasmid multilocus sequence type (pMLST), and resistance gene profiles using the Bacterial Analysis Pipeline-Batch Upload from the Center for Genomic Epidemiology (CGE; http://www.genomicepidemiology.org). Kmer-Finder (37) was used to confirm MALDI-TOF species identification and SISTR (38) for *Salmonella* serovar confirmation. Mapping of raw reads against the PacBio bacterial chromosome sequence of the meat-derived *bla*_VIM-1_-harboring S. Infantis isolate 15-SA01028 (GenBank accession number CP026660.1) (8) and subsequent SNP analysis of *Salmonella* isolates were carried out with the BioNumerics software (v7.6; Applied Maths, Ghent, Belgium). The *bla*_VIM-1_-negative *S*. Infantis isolate 09-03100 was included in SNP analysis, as this isolate represents the closest relative among the investigated isolates from the national strain collection of the NRL Salmonella (14). In terms of plasmid sequence comparison, raw reads were mapped against the PacBio sequence of pSE15-SA01028 (GenBank accession number CP026661.1), using CLC Genomics workbench 9.5. Visualization of differences in plasmid backbones was performed with BLAST Ring Image Generator 0.95 (BRIG) (39). The absence of the *bla*_VIM-1_-harboring Tn*21*-like transposon in the plasmid sequence of isolate N2-6 was confirmed by plasmid closure using PCR, followed by sequencing as previously described (35).

### Data availability.

Whole-genome raw sequence data of all *bla*_VIM-1_-positive isolates and the *S*. Infantis *bla*_VIM-1_-negative isolate 09-03100 were submitted to the ENA database under the following accession numbers: for N2-1, ERS2958092; N2-2, ERS2958093; N2-3, ERS2958094; N2-4, ERS2958095; N2-5, ERS2958096; N2-6, ERS2958097; N2-8, ERS2958098; R3, ERS2154041; R25, ERS2958099; R27, ERS2958100; V363, ERS2958101; G-336-1a, ERS2101552; 15-SA1028, ERS2488743; 16-SA00749, ERS2488744; and 09-03100, ERS2958102.

10.1128/mSphere.00089-19.3 FIG S3 S1 PFGE analysis of all six *Salmonella* isolates detected in this study (N2-1 to N2-6) and the Enterobacter cloacae isolate N2-8. Br, molecular size standard *Salmonella* Braenderup strain H9812 (restricted with XbaI). An asterisk marks strains positive after *bla*_VIM-1_ hybridization. Download FIG S3, PDF file, 0.1 MB. Copyright © 2019 Roschanski et al. 2019 Roschanski et al. This content is distributed under the terms of the Creative Commons Attribution 4.0 International license.
