# Supplementary figures and images for: Detection of VIM-1-Producing Enterobacter cloacae and Salmonella enterica Serovars Infantis and Goldcoast at a Breeding Pig Farm in Germany in 2017 and Their Molecular Relationship to Former VIM-1-Producing S. Infantis Isolates in German Livestock Production
Source: mSphere. 2019 Jun 12;4(3):e00089-19. doi: 10.1128/mSphere.00089-19 (PMC6563352; doi:10.1128/mSphere.00089-19)

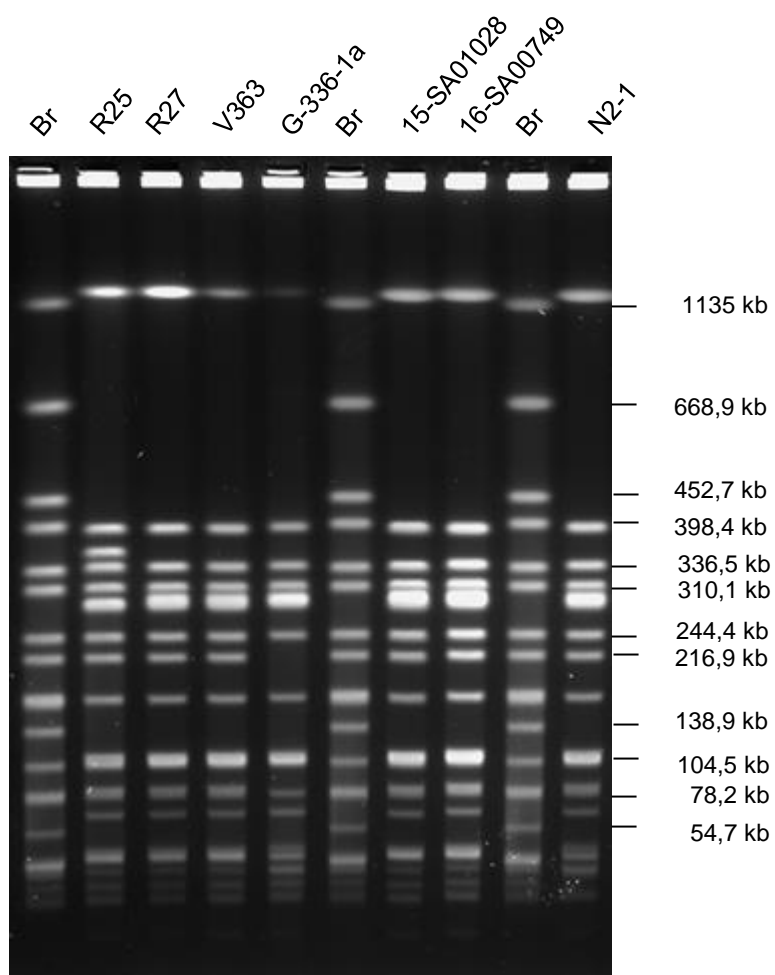

Supplement: FIG S1 [file mSphere.00089-19-sf001.pdf]

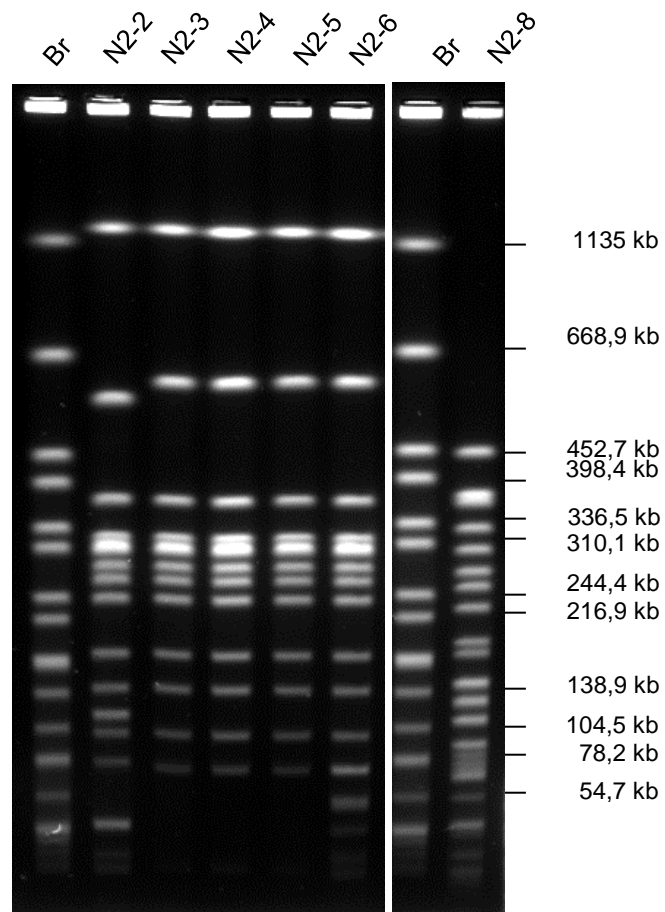

Supplement: FIG S2 [file mSphere.00089-19-sf002.pdf]

|            | G336-1a | R27 | R3 | V363 | R25 | 15-SA01028 | N2-1 | 16-SA00749 | 09-03100 |
|------------|---------|-----|----|------|-----|------------|------|------------|----------|
| G336-1a    | 0       |     |    |      |     |            |      |            |          |
| R27        | 6       | 0   |    |      |     |            |      |            |          |
| R3         | 6       | 0   | 0  |      |     |            |      |            |          |
| V363       | 6       | 0   | 0  | 0    |     |            |      |            |          |
| R25        | 18      | 12  | 12 | 12   | 0   |            |      |            |          |
| 15-SA01028 | 47      | 40  | 41 | 40   | 35  | 0          |      |            |          |
| N2-1       | 47      | 41  | 41 | 41   | 35  | 28         | 0    |            |          |
| 16-SA00749 | 51      | 45  | 45 | 45   | 39  | 38         | 38   | 0          |          |
| 09-03100   | 103     | 97  | 97 | 97   | 89  | 104        | 104  | 108        | 0        |

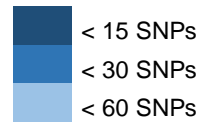

Supplement: TABLE S2 [file mSphere.00089-19-st002.pdf]

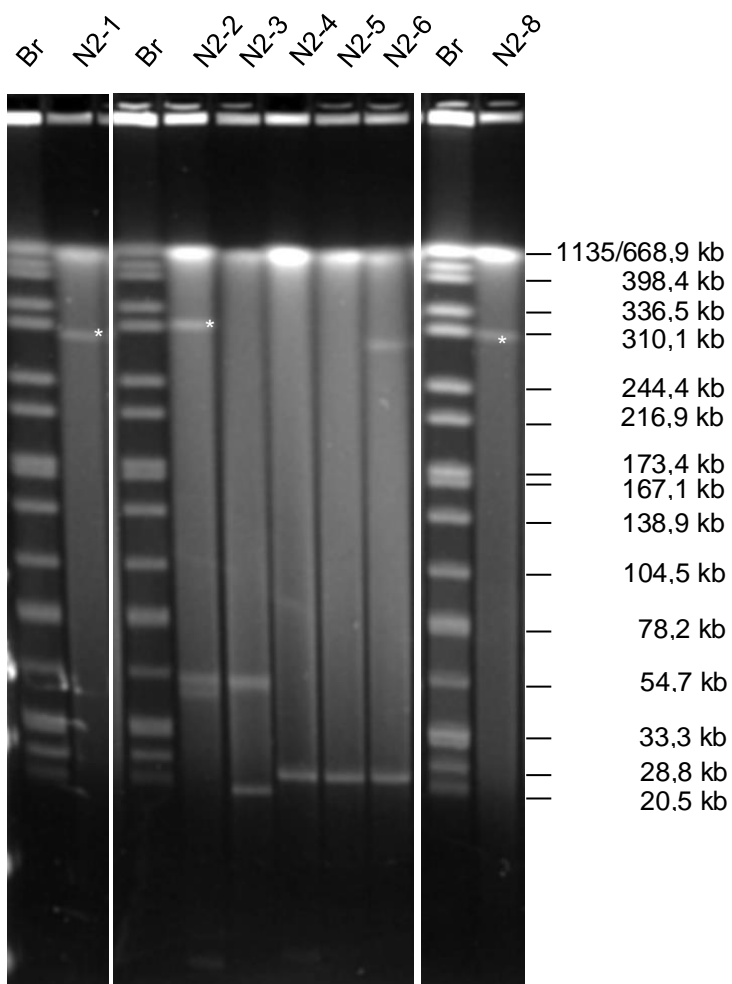

Supplement: FIG S3 [file mSphere.00089-19-sf003.pdf]
