# Supplementary material for: Detection of VIM-1-Producing Enterobacter cloacae and Salmonella enterica Serovars Infantis and Goldcoast at a Breeding Pig Farm in Germany in 2017 and Their Molecular Relationship to Former VIM-1-Producing S. Infantis Isolates in German Livestock Production
Source: mSphere. 2019 Jun 12;4(3):e00089-19. doi: 10.1128/mSphere.00089-19 (PMC6563352; doi:10.1128/mSphere.00089-19)
Supplement: TABLE S1 [file mSphere.00089-19-st001.pdf]

Stable	No. of pooled feces samples (pooled units)	No. of boot swabs (pooled units)
Gestation stall 1		13 (2 pens)
Gestation stall 2		14 (2 pens)
Gestation stall 3		12 (2 pens)
Gestation stall 4	4 (2 pens)	3 (2 pens)
Breeding center 1	3 x (~8 breeding pens)	
Breeding center 3	4 x (~8 breeding pens)	
Ferrowing barn 1	1 (~25 ferrowing pens)	1 (central corridor)
Ferrowing barn 2	1 (~25 ferrowing pens)	1 (central corridor)
Ferrowing barn 3	1 (~25 ferrowing pens)	1 (central corridor)
Ferrowing barn 4[a]	1 (~25 ferrowing pens)	1 (central corridor)
Ferrowing barn 5	1 (~25 ferrowing pens)	1 (central corridor)
Rearing quarter 1[b]	6 (6 pens) + 5 (12 pens)	1 (central corridor)
Rearing quarter 2	7 (4 pens)	1 (central corridor)
Rearing quarter 3	4 (5 pens)	1 (central corridor)
Rearing quarter 4	9 (6 pens)	1 (central corridor)
**Total number:**	**47**	**51**
**Additional samples:** Two boot swabs derived from the central corridor and one sample was taken from each of the three liquid manure pits.		

[a] area positive for $bla_{\text{VIM-1}}$ harboring *S.* Goldcoast
[b] area positive for $bla_{\text{VIM-1}}$ harboring *S.* Infantis and *E. cloacae*
